# Supplementary material for: Modeling the t(2;5) Translocation of Anaplastic Large Cell Lymphoma Using CRISPR-Mediated Chromosomal Engineering
Source: Cancers (Basel). 2025 Jul 2;17(13):2226. doi: 10.3390/cancers17132226 (PMC12249153; doi:10.3390/cancers17132226)

Supplementary data

Tables

Suppl. Table S1: sgRNAs tested for induction of the *Npm1-Alk* translocation

Suppl. Table S2: List of RPA3 sgRNA sequences

Suppl. Table S3: List of all mutations observed in M-FISH of Ba/F3 cells overexpressing plasmid Npm-Alk and BaF3 cells after induction of NPM-ALK translocation

Figures

Suppl. Figure S1: Flow cytometric monitoring of Ba/F3_RIEP cells after doxycycline induced targeting of essential genes using the inducible CRISPR/Cas9 system.

Suppl. Figure S2: Flow cytometric monitoring of cells targeting of essential genes using the constitutively active CRISPR/Cas9 system (Cas9 and eCas9)

Suppl. Figure S3: Schematic outline of the syntenic chromosomal murine regions on chromosomes 11 and 17 corresponding to the human NPM and ALK gene loci on chromosomes 2 and 5

Suppl. Figure S4: Schematic workflow of CRISPR/Cas9 induced Npm1-Alk translocation in Ba/F3 cells

Suppl. Figure S5: Schematic workflow of CRISPR/Cas9 induced Npm1-Alk translocation in Ba/F3 cells with a dual sgRNA delivery system.

Suppl. Figure S6: Schematic of CRISPR/Cas9 engineered NPM1-ALK translocation compared to endogenous NPM1-ALK translocation in human ALCL

Suppl. Figure S7: PCA analysis of mRNA expression in Ba/F3 cells (control) compared to Ba/F3 cells overexpressing the Npm-Alk cDNA (MIG) and Ba/F3 cells transformed by CRISPR-mediated Npm-Alk translocation (eCAS9)

Suppl. Figure S8: Heatmap: Log2 fold Change in gene expression pattern in Ba/F3 cells (control) compared to Ba/F3 cells overexpressing the Npm-Alk cDNA (MIG) in comparison to transformed Ba/F3 by CRISPR-mediated Npm-Alk translocation (eCAS9)

Suppl. Figure S9: Gene set enrichment analysis (GSEA) of differentially expressed genes located on chromosomal regions flanking the breakpoint sites of NPM1 and ALK in Ba/F3 cells

Suppl. File S1: Collection of uncropped Western Blots and Electrophoresis Gels

Tables

Supplementary Table S1:

sgRNAs tested for induction of the *Npm1-Alk* chromosomal translocation

sgRNA No.	*Npm1*	*Alk*
1	5´- GTATCTTTGCACATCTGCCA-3´	5´- GTGAACCAAAGGAAACCCTT-3´
2	5´- GGTCAGACAAGACAATTTCC-3´	5´- AGAAGCATGCAGGCAGACTC-3´
3	5´- GATTTCTGTCTTTAAAATAC-3´	5´- GTGTATATGTGGCTCAAGCC-3´

The shown sgRNAs (No.1-3) were designed with E-CRISPR (43)

Supplementary table S2:

List of RPA3 sgRNA sequences

No.	Plasmid name	Sequence of sgRNA
1	GBAC_Rpa3e1.1	5´-ACGGGCCGGTCGATATACTG-3´
2	GBAC_Rpa3e1.2	5´-GACCGGCCCGTGTGCTTCGT-3´
3	GBAC_Rpa3e1.3	5´-GCTGGCGTTGACGCGCGCTT-3´

sgRNAs were reported to target efficiently (56)

Supplementary Table S3:

List of all mutations observed in M-FISH of Ba/F3 cells overexpressing plasmid Npm-Alk and BaF3 cells after induction of NPM-ALK translocation

Cells	Mutations per Sample
Ba/F3	• 80,XX. der(3)t(3;6),der(8)t(2;8),der(8)t(2;8),der(8)t(2;8),-14,+19,+19, -X,-X<br>• 78,XX. t(2;8),der(3)t(3;6),der(3)t(3;6),+6,der(8)t(2;8),-13,-14,-16,+19,+19, -X,-X<br>• 81,XX. der(3)t(3;6),der(3)t(3;6),+6,der(8)t(2;8),der(8)t(2;8),-14,+17,+19,+19, -X,-X<br>• 80,XX. t(2;8),t(2;8),der(3)t(3;6),der(3)t(3;6),+8,-14,+15,+19,+19, -X,-X
Ba/F3_MNAIE	• 79,XX.

	dic(1;12),+dic(1;12),der(3)t(3;6),der(3)t(3;6),der(8)t(2;8),-12,-14,+19,+19,-X,-X<br>● 78,XX. der(3)t(3;6),der(3)t(3;6),der(8)t(2;8),der(8)t(2;8),-12,dup(14),-14,+19,+19,-X,-X<br>● 79,XX. t(2;8),t(2;8),der(3)t(3;6),der(3)t(3;6),-14,+19,+19,-X,-X<br>● 78,XX. t(2;8),der(3)t(3;6),der(3)t(3;6),der(8) t(2;8),-14,+19,+19,-X,-X
Ba/F3_MSCV_Cas9_Puro_ GBAC_Npm1_1_ GBACy_Alk_1	● 75,XX. t(2;8),der(3)t(3;6),der(3)t(3;8),-6,der(8)t(2;8),-11,-14,der(17)**t(11;17)**,-17,+19,-X,-X<br>● 79,XX. t(2;8),t(2;8),+2,der(3)t(3;6),dic(3;9),+del(3),-6,+der(8)t(2;8),-11,-14,der(17)**t(11;17)**,-17,+19,+19,-X,-X<br>● 77,XX. der(3)t(3;6),der(3)t(3;8),+dic(3),-6,der(8)t(2;8),der(8)t(2;8),-11,-14,der(17)**t(11;17)**,-17,+19,+19,-X,-X<br>● 75,XX. der(3)t(3;6),der(3)t(3;9),-6,der(8)t(2;8),der(8)t(2;8),-9,-11,-14,der(17)**t(11;17)**,-17,+19,+19,-X,-X
Ba/F3_MSCV_Cas9_Puro_ 2GBAC_Npm1-Alk	● 77,XX. 1,t(2;8),t(2;8),der(3)t(3;6),der(3)t(3;6),der(3)t(3;18),rob(3;9), **t(11;17)**,-12,-14,+19,+19,-X-X<br>● 77,XX. der(3)t(3;6),der(3)t(3;6),der(3)t(3;18),der(8)t(2;8),der(8)t(2;8),rob(3;9),**t(11;17)**,-12,-14,+19,-X-X<br>● 78,XX. t(2;8),t(2;8),der(3)t(3;6),der(3)t(3;6),der(3)t(3;11),rob(3;9), **t(11;17)**,12,-14,+19,+19,-X-X<br>● 75,XX.ish t(2;8),t(2;8),der(3)t(3;6),der(3)t(3;6),der(3)t(3;18),rob(3;9), **t(11;17)**,-12,-14,-15,-X-X

Karyotyping of the genome of Ba/F3 cells with no infection (1st row), overexpressing plasmid Npm1-Alk (2nd row), infected with a Cas9 vector and double infected with sgRNA vectors (3rd row) and infected with a Cas9 vector and a single infection of dual sgRNA vector (4th row). Shown are one out of four assays performed each.

These experiments were performed by Martina Auer, Michael Speicher, Institute of Human Genetics, Medical University of Graz

Supplementary Figures

Supplementary Figure S1:

Flow cytometric monitoring of Ba/F3_RIEP cells after doxycycline induced targeting of essential genes (RPA3) using the inducible CRISPR/Cas9 system

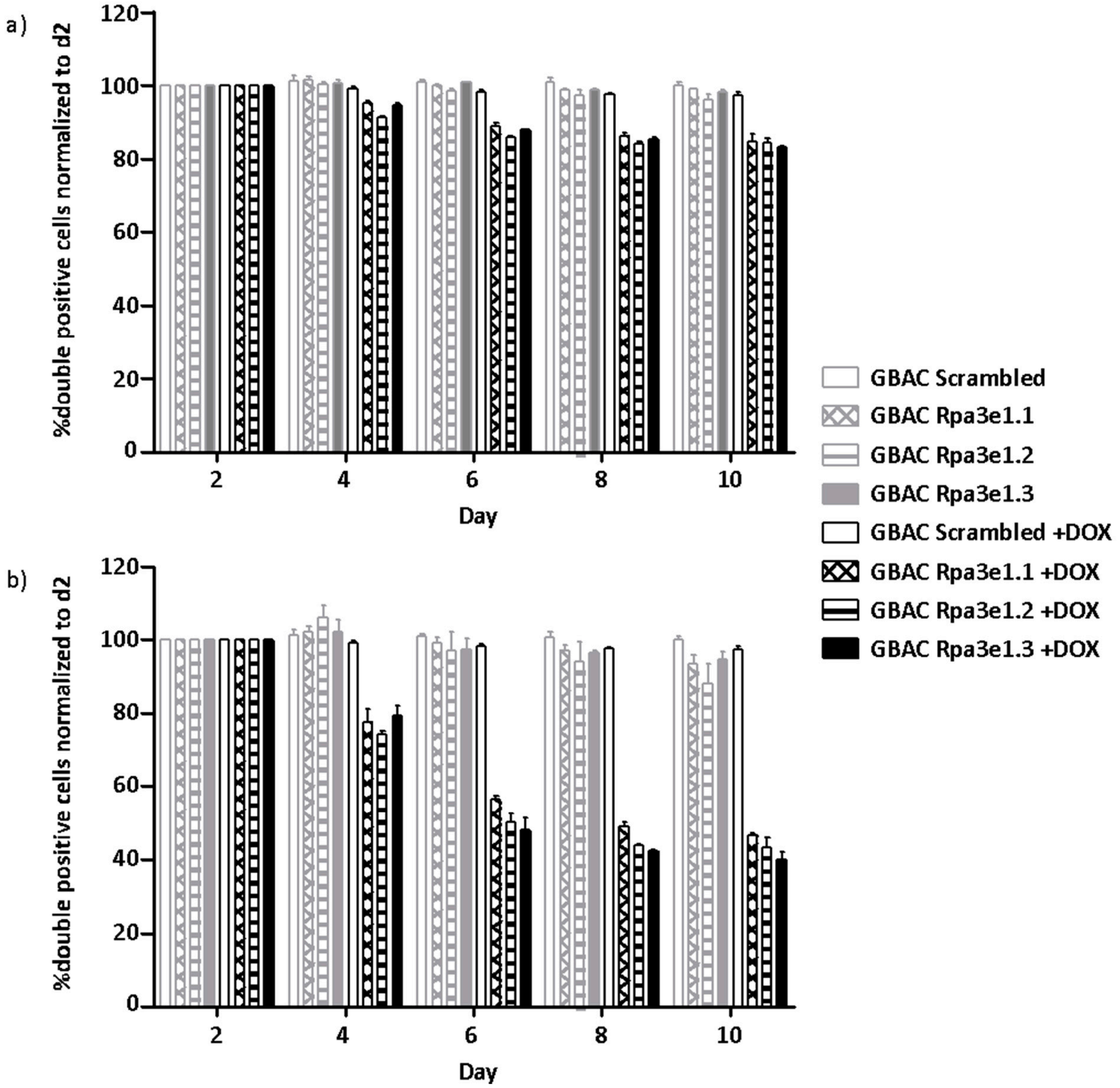

Cell viability of GFP and Cherry positive cells was monitored every two days post infection. a) Post GBAC infection, blasticidine S was used for selection until GFP and Cherry positive cells accounted for over 90%.

b) Post GBAC infection, no further selection was used.

All experiments were performed in biological triplicates.

For the sake of more clarity significance tests were not marked with * (p<0,05), ** (p<0,01) or *** (p<0,001). All doxycycline treated groups (selected and unselected) demonstrated a p-value of p<0,001 when compared to their treated control group (Scrambled +Dox) or their respective untreated group (Rpa3e1.1, Rpa3e1.2 or Rpa3e1.3) on day 4,6,8 and 10.

Supplementary Figure S2

Flow cytometric monitoring of cells targeting of essential genes using the constitutively active CRISPR/Cas9 system (Cas9 and eCas9)

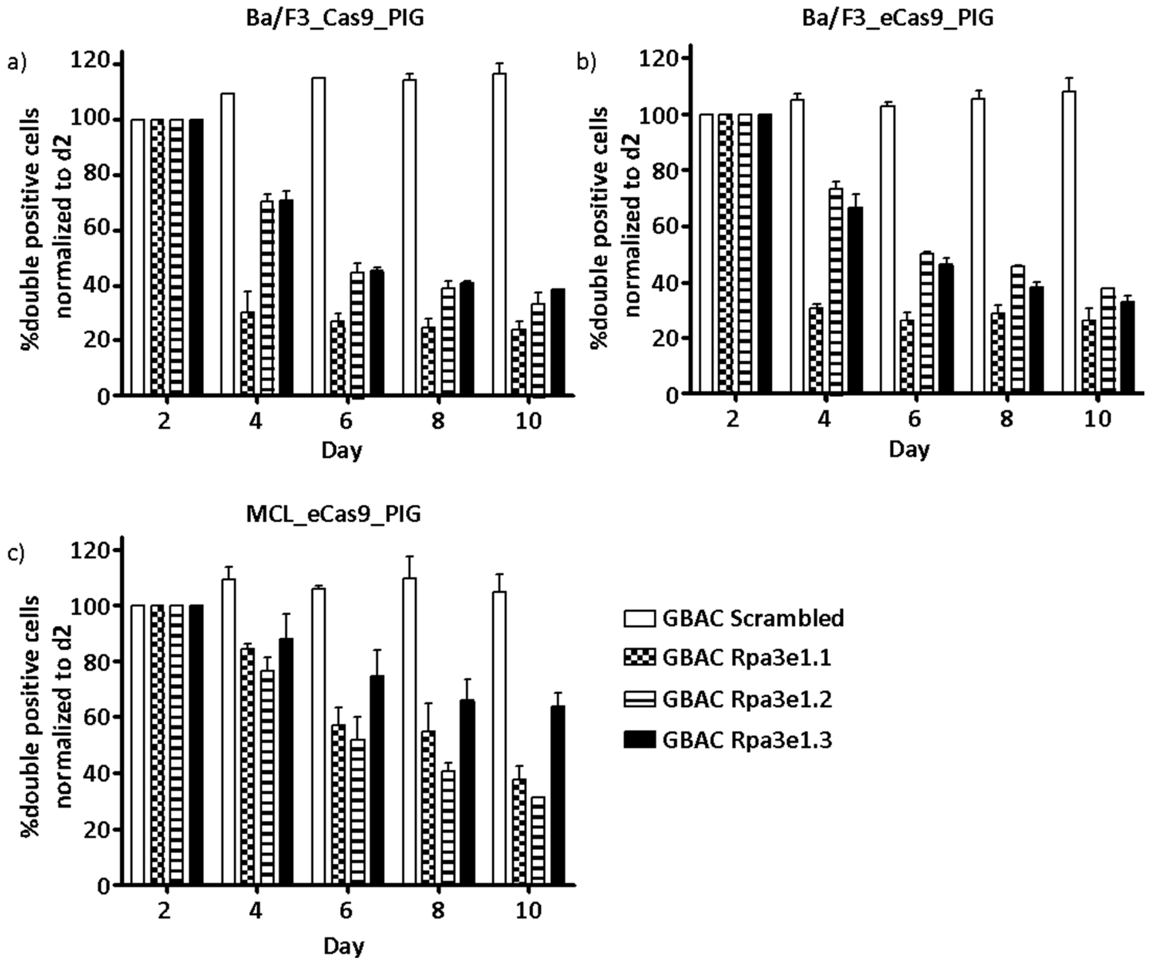

Ba/F3 cells were either infected with Cas9_PIG (a) or eCas9_PIG (b). MCL 22-1 cells were infected with eCas9_PIG (c). Cell viability of GFP and Cherry positive cells was monitored every 2d post infection.

All experiments were performed in biological duplicates.

For the sake of clarity significance tests were not marked with * (p<0,05), ** (p<0,01) or *** (p<0,001). All groups in Ba/F3 cells (a and b) irrespective of the used Cas9 variant demonstrated a p-value of p<0,001 when compared to their control group (GBAC Scrambled) on day 4,6,8 and 10. The groups in c) demonstrated a p-value of p<0,01 when using GBAC Rpa3e1.1 and GBAC Rpa3e1.3 only on day 4. All other timepoints for every group were demonstrating a p-value of p<0,001 including GBAC Rpa3e1.2 on day 4.

Supplementary Figure S3:

Schematic outline of the syntenic chromosomal murine regions (outer ring) on chromosomes 11 (NPM1, left) and 17 (ALK, right) corresponding to the human NPM and ALK gene loci on chromosomes 2 and 5 (inner ring).

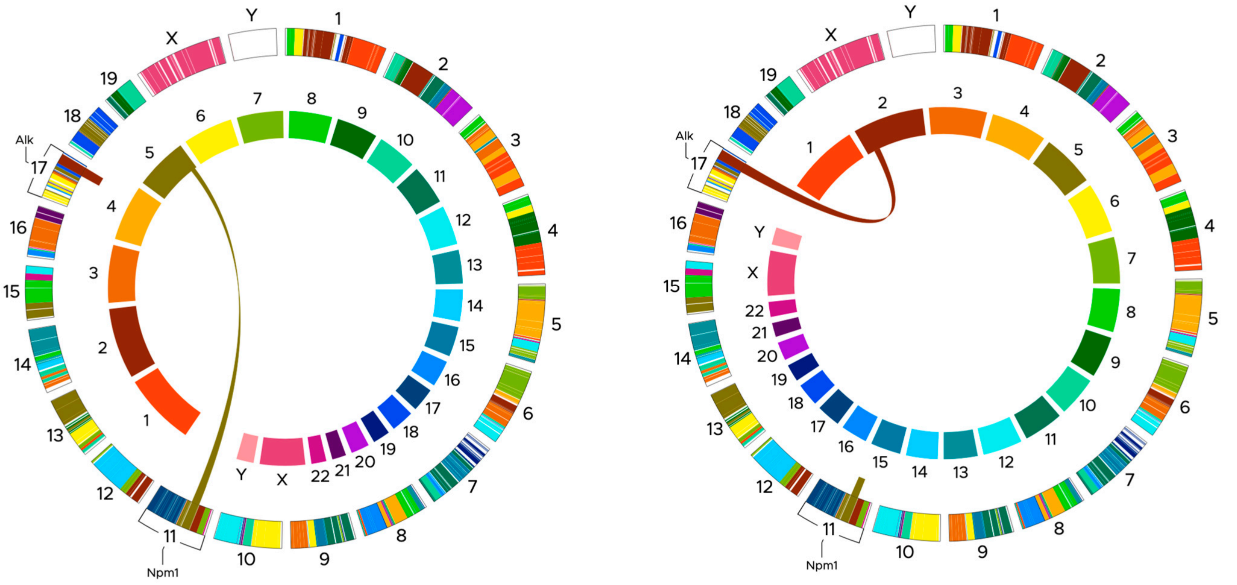

Supplementary Figure S4:

Schematic workflow of CRISPR/Cas9 induced *Npm1-Alk* translocation in Ba/F3 cells.

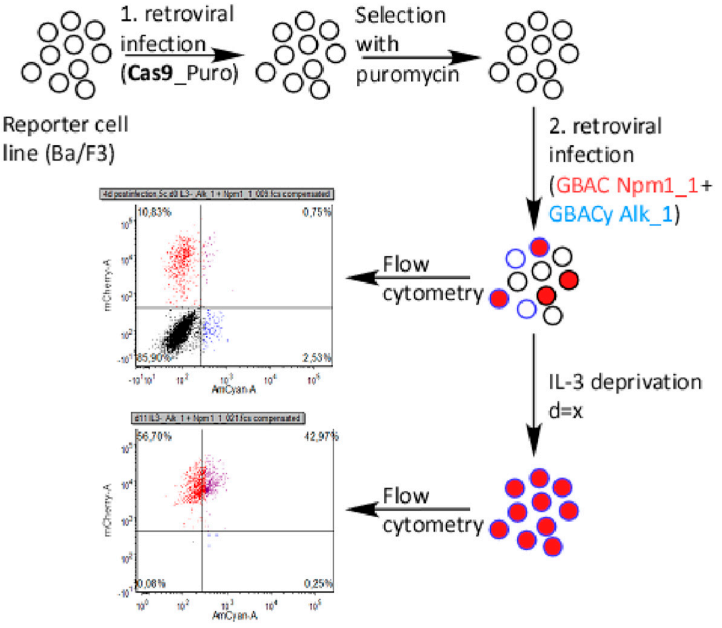

Supplementary Figure S5:

Schematic workflow of CRISPR/Cas9 induced *Npm1-Alk* translocation in Ba/F3 cells with a dual sgRNA delivery system.

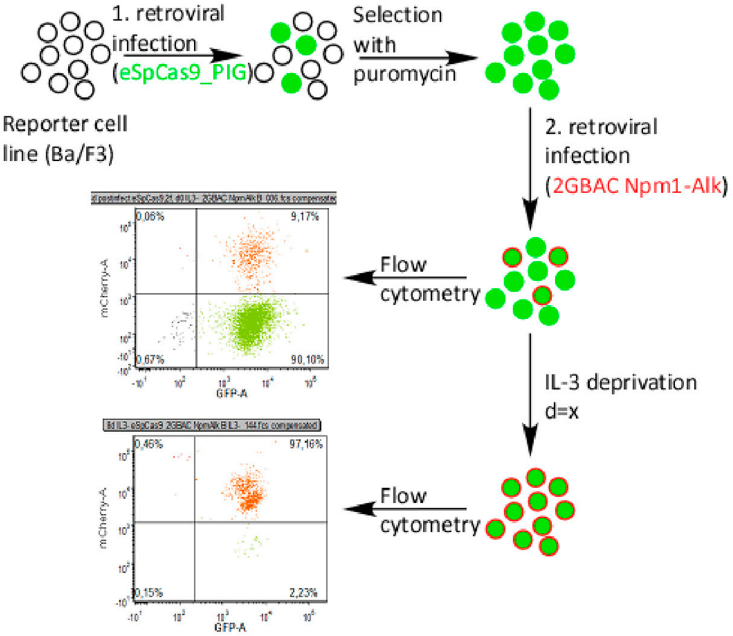

Supplementary Figure S6:

Schematic of CRISPR/Cas9 engineered NPM1-ALK translocation compared to endogenous NPM1-ALK translocation in human ALCL.
The NPM1 gene (12 exons) and the ALK gene (29 exons) are highly conserved between humans and mice. The translocation t(2;5)(p23;q35) fuses exon 4 of NPM1 to exon 20 of ALK, with the breakpoint of NPM1 predominantly located within intron 4 encompassing a region of approximately 1 kilobase, and the breakpoint of ALK typically occurring within intron 19 spanning about 2,2 kilobases.

CRISPR/Cas engineerend NPM1-Alk Translocation in murine Ba/F3 cells9

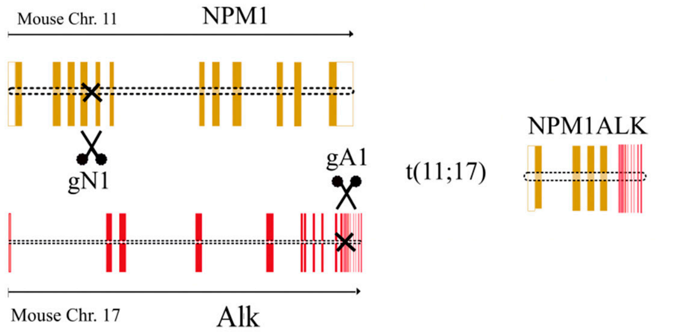

Endogenous NPM1-ALK Translocation in Human ALK-Positive Anaplastic Large Cell Lymphoma

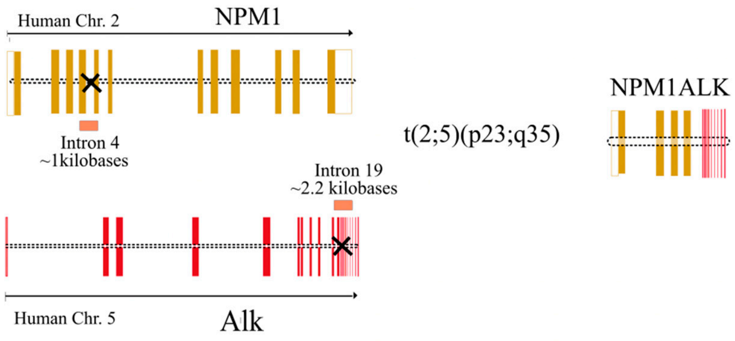

Supplementary Figure S7:
Principle component analysis (PCA) of samples of Ba/F3 cells transformed by NPM-ALK translocation (eCAS9), by overexpression of the NPM-ALK fusion gene (MIG) or Ba/F3 control cells growing in the presence of IL-3.

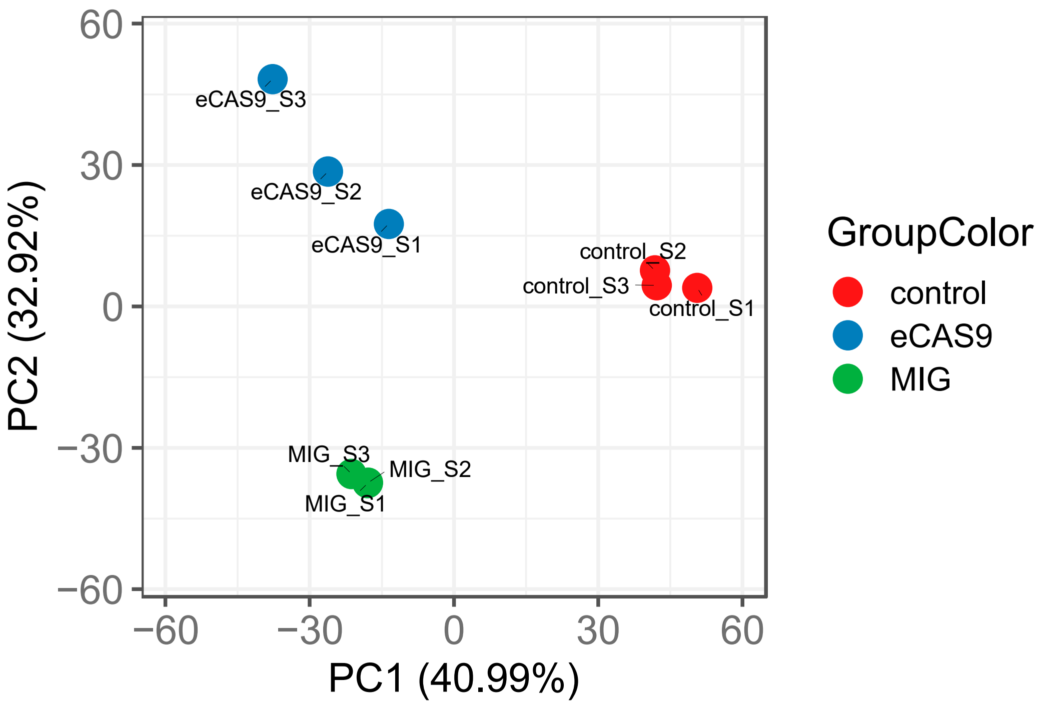

Supplementary Figure S8:
Heatmap**:** Log2 fold Change in gene expression pattern in Ba/F3 cells (control), Ba/F3 cells overexpressing the *Npm-Alk* cDNA (MIG) or transformed Ba/F3 by CRISPR-mediated *Npm-Alk* translocation (eCAS9).

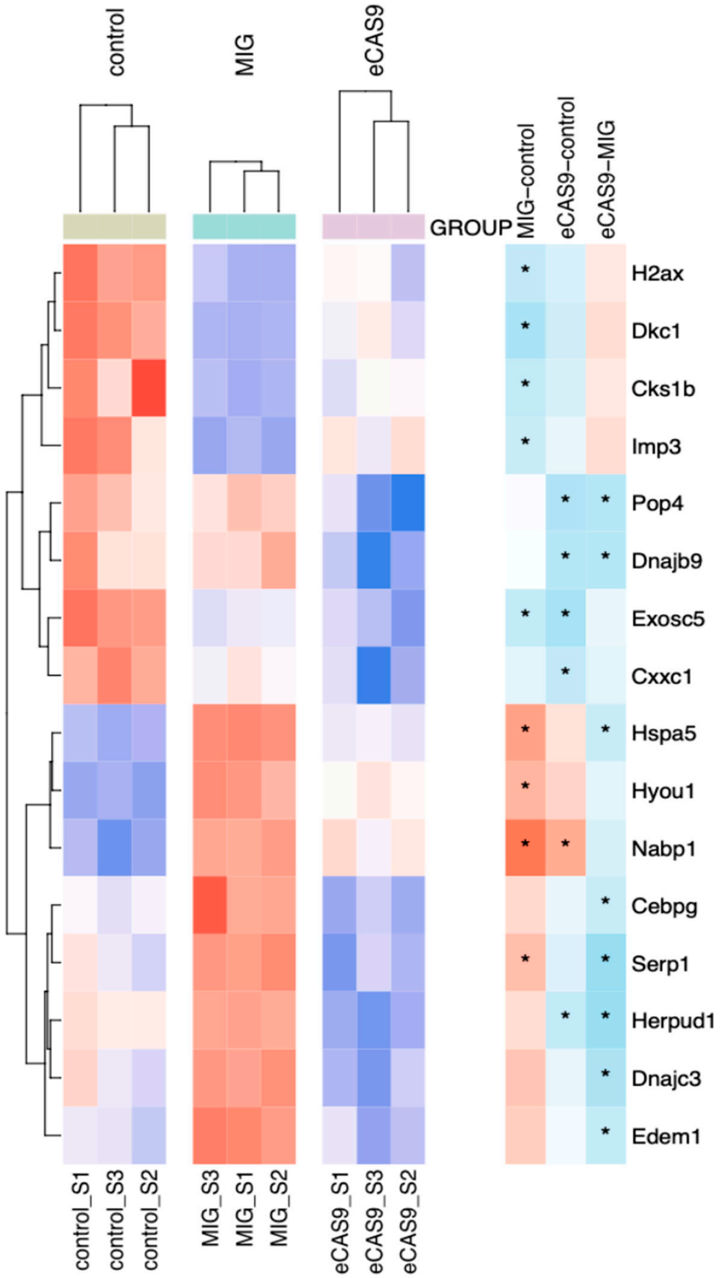

Supplementary Figure S9:

Gene set enrichment analysis (GSEA) of differentially expressed genes located on chromosomal regions flanking the breakpoint sites of NPM1 and ALK in Ba/F3 cells overexpressing NPM-ALK cDNA versus Ba/F3 cells carrying a t(11;17) translocation. Only a small set of genes 10 Mb downstream of the NPM1 breakpoint were upregulated, whereas most of the gene sets adjacent to the NPM1 and ALK locus within 10 or 100Mb showed significantly decreased expression.

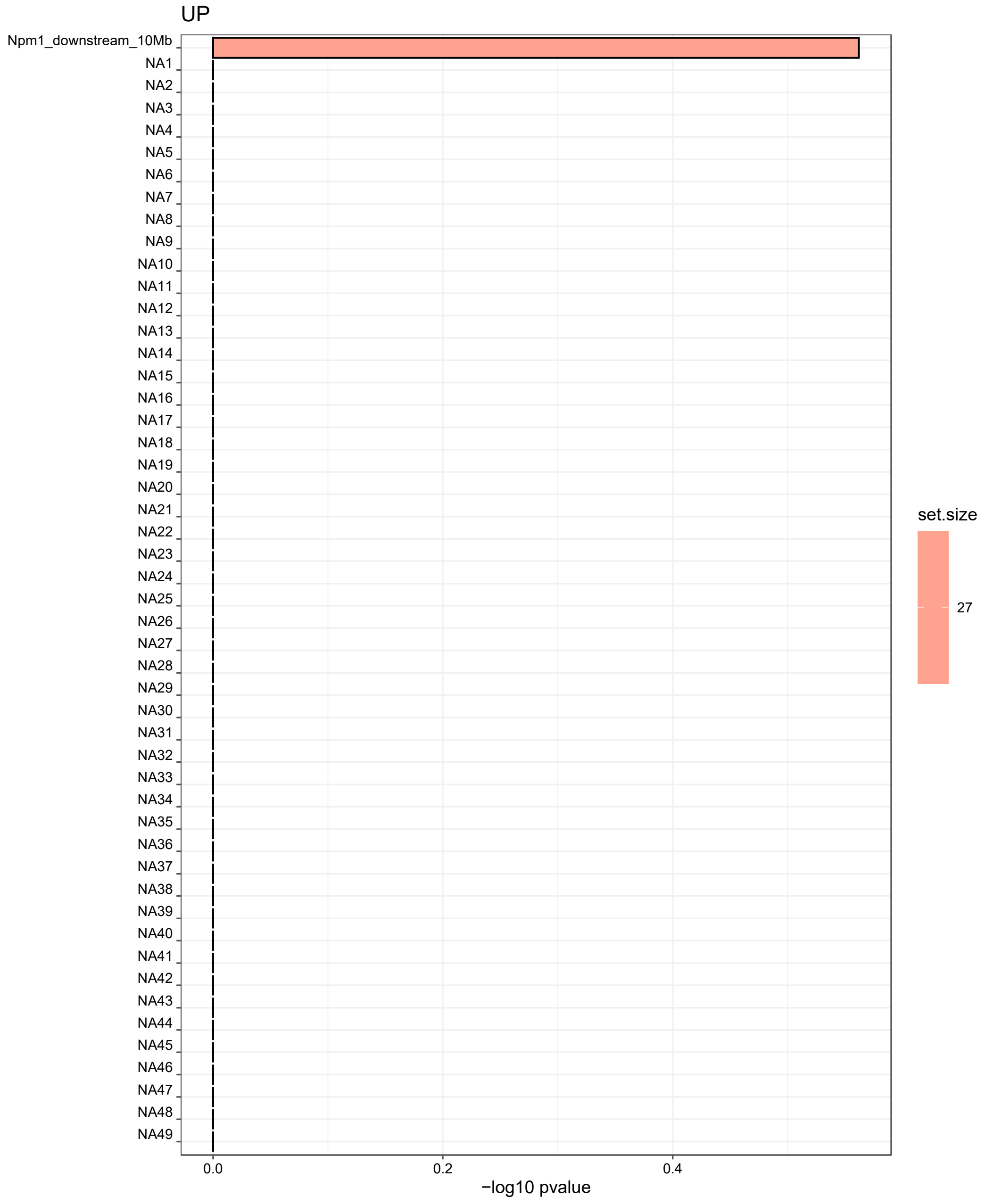

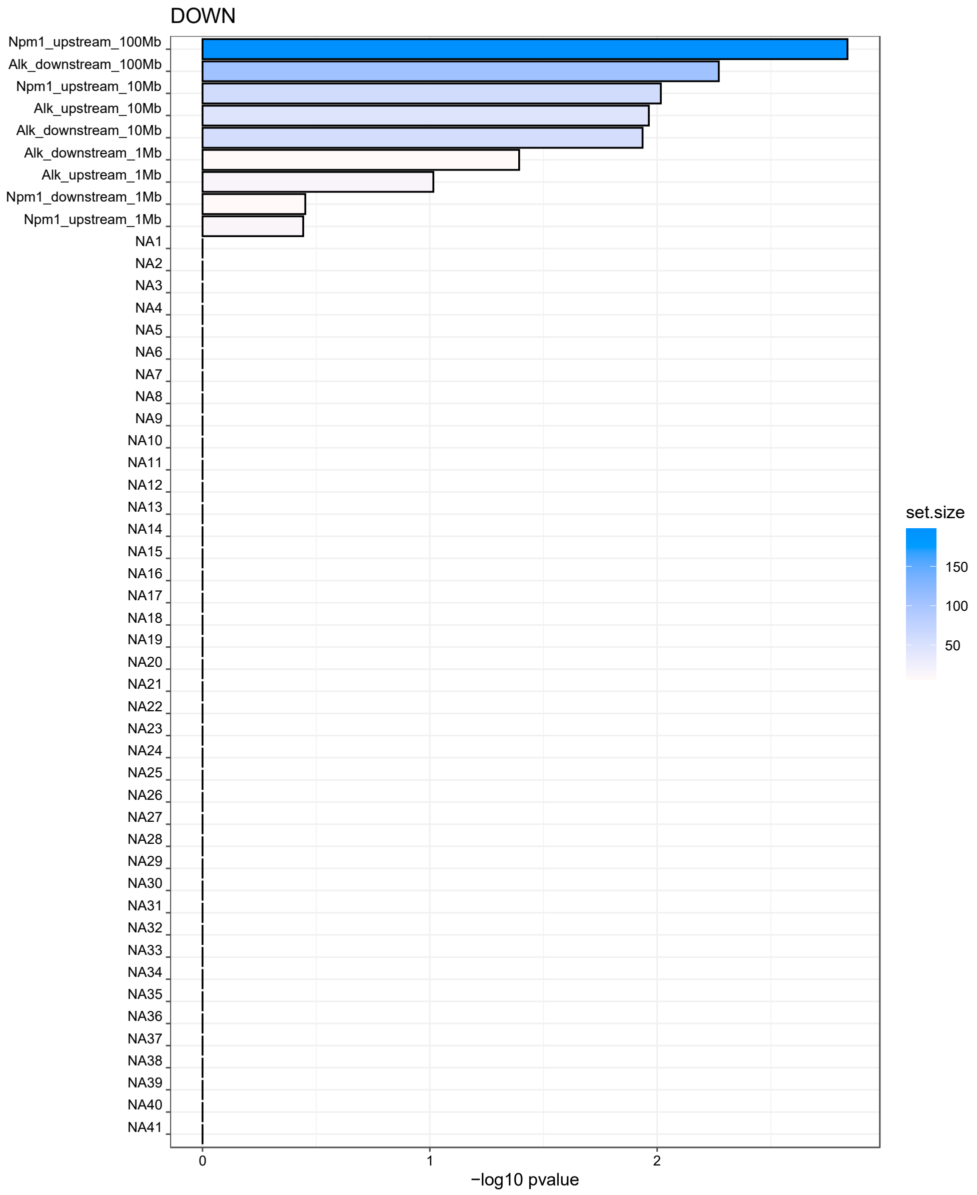
DOWN
Npm1_upstream_100Mb
Alk_downstream_100Mb
Npm1_upstream_10Mb
Alk_upstream_10Mb
Alk_downstream_10Mb
Alk_downstream_1Mb
Alk_upstream_1Mb
Npm1_downstream_1Mb
Npm1_upstream_1Mb
NA1
NA2
NA3
NA4
NA5
NA6
NA7
NA8
NA9
NA10
NA11
NA12
NA13
NA14
NA15
NA16
NA17
NA18
NA19
NA20
NA21
NA22
NA23
NA24
NA25
NA26
NA27
NA28
NA29
NA30
NA31
NA32
NA33
NA34
NA35
NA36
NA37
NA38
NA39
NA40
NA41
0
1
2
−log10 pvalue
set.size
150
100
50

Supplementary Materials File S1

Western Blot

Figure 1C

Cas9 expression levels: Western Blot (WB) analysis of Ba/F3, HL-60, K562 cells infected with RIEP and T3GCasVIN after 2d of doxycycline treatment and Cas9 expression in Ba/F3 with constitutive Cas9 expression with C_P and eC_PIG vectors.

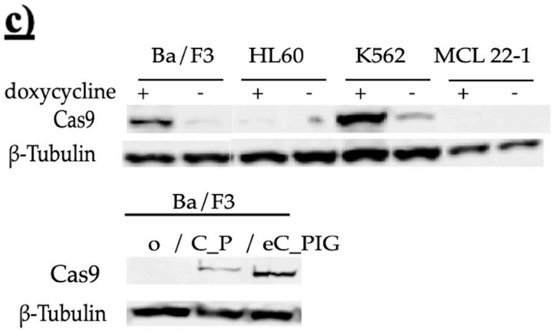

	Normalized densitometry analysis (Cas9/tubulin)
Ba/F3 +	0,652
Ba/F3 -	0,332
HL60 +	0,016
HL60 -	0,088
K562 +	0,844
K562 -	0,160
MCL 22-1+	0,014
MCL 22-1 -	0
Ba/F3 o	0
Ba/F3 C_P	0,033
Ba/F3 PIG	0,247

Figure 1c

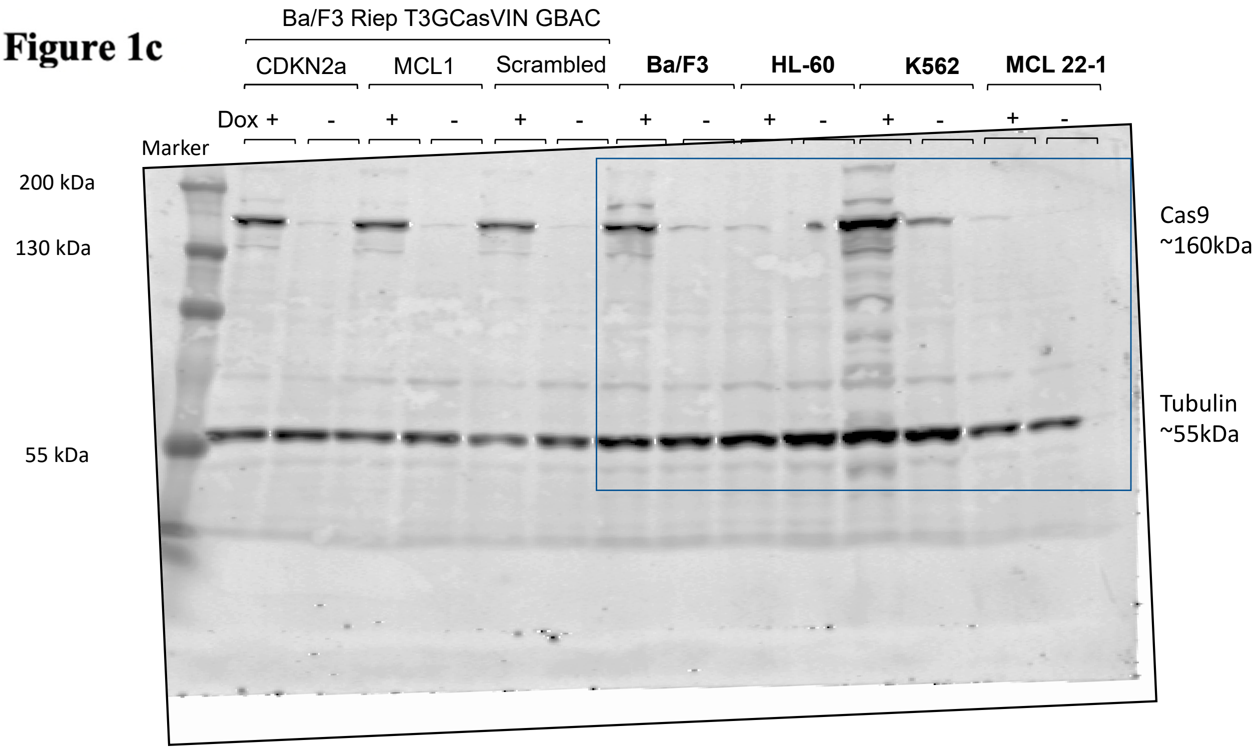

Figure 1c

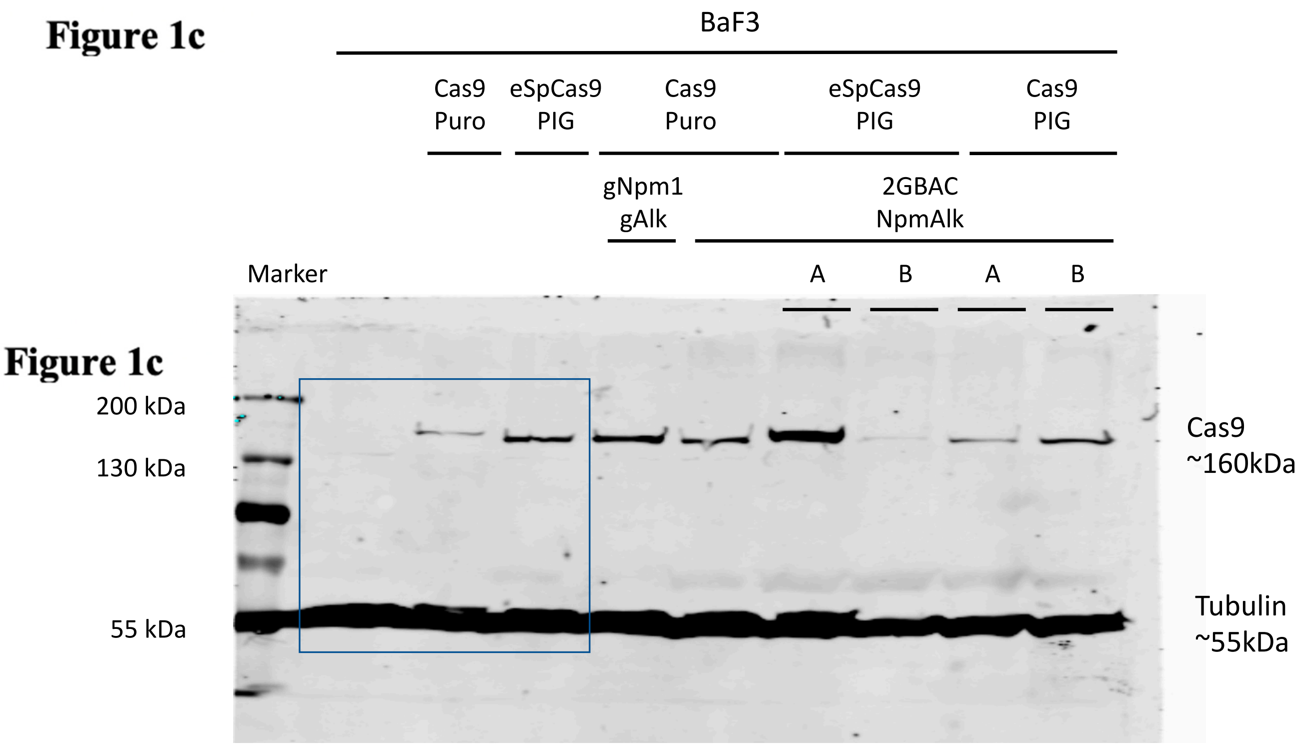

Figure 1c

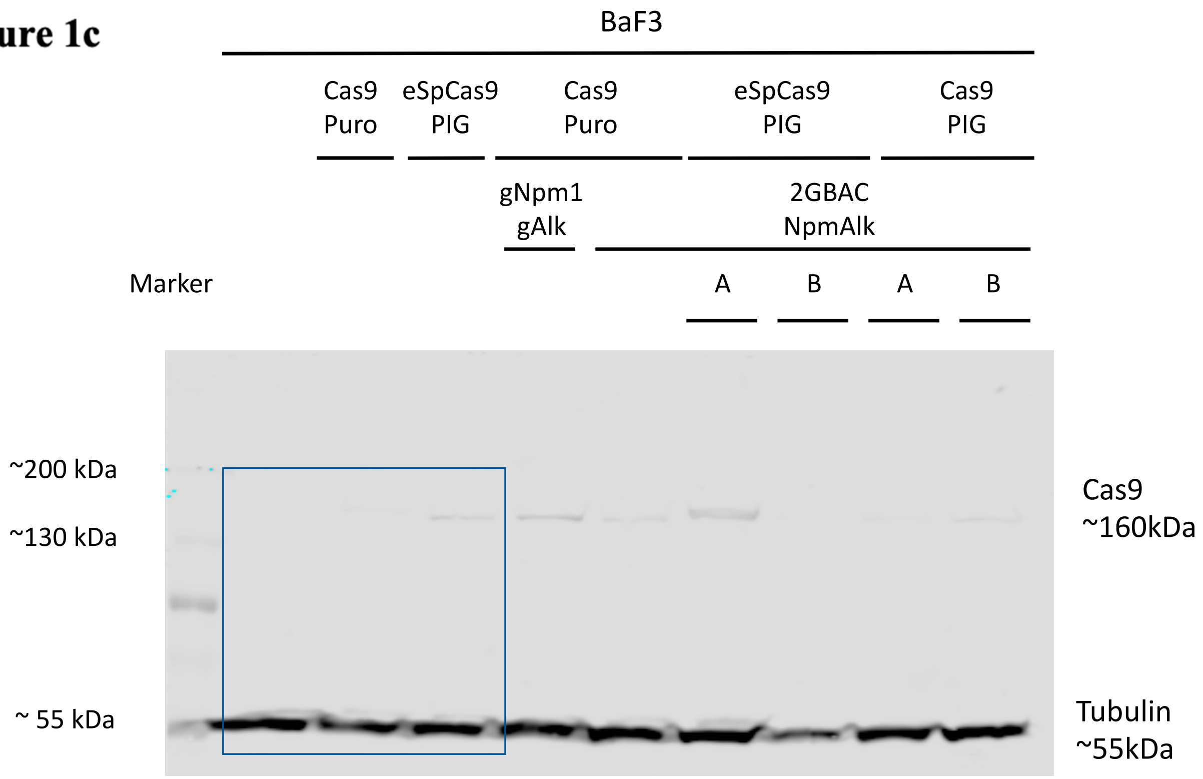

Figure 2c

Alk western blot of several constitutive Cas9 constructs with or without sgRNAs in Ba/F3 cells. β-tubulin control

	Normalized densitometry analysis (ALK/tubulin)
Contr.	0,005
C_P	0,002
eC-PIG	0,008
C_P gN1+gA1	1,390
C_P 2gN1A1	1,360
eC-PIG 2gN1A1	0,99
eC-PIG 2gN1A1	0,295
C-PIG 2gN1A1	0,397
C-PIG 2gN1A1	0,932

Figure 2 c

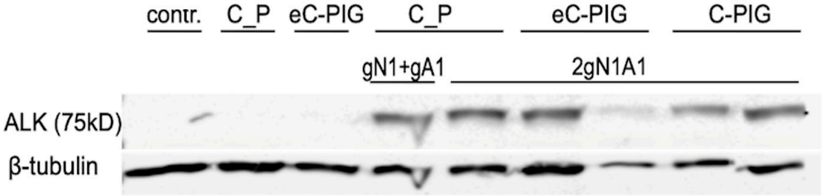

Figure 2 c

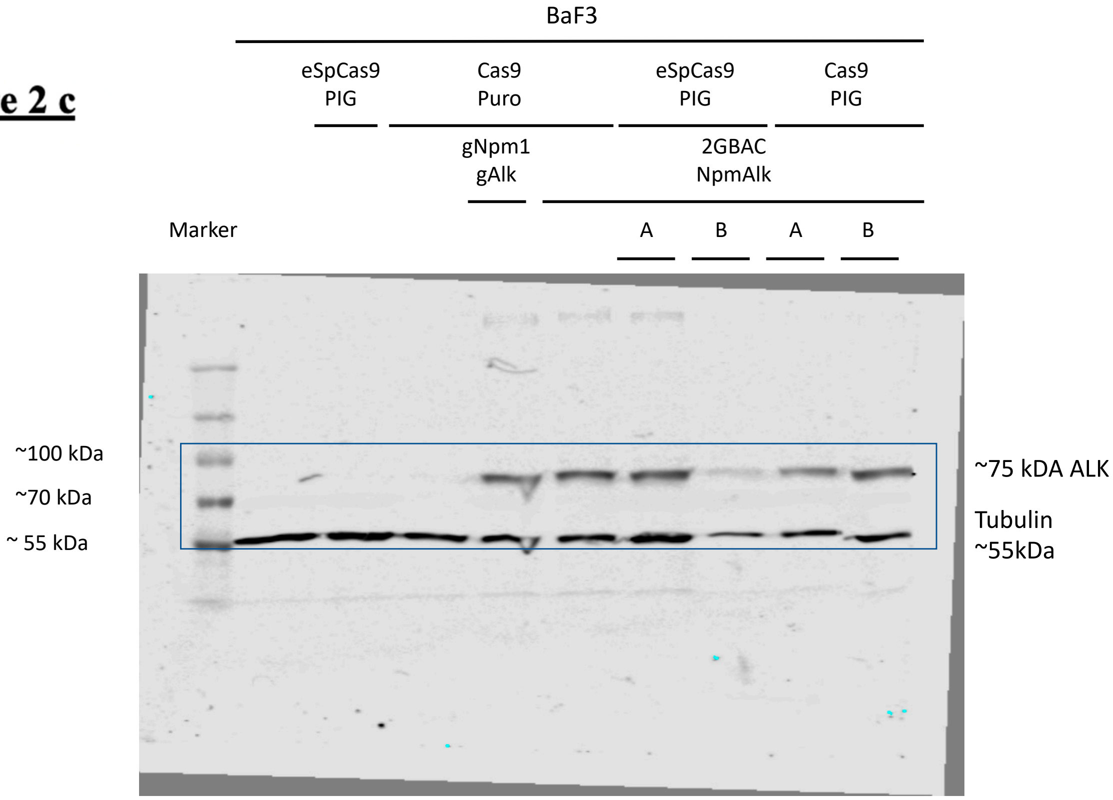

Figure 2d
Gel electrophoresis

PCR of the resulting Npm1-Alk translocation from different constructs.

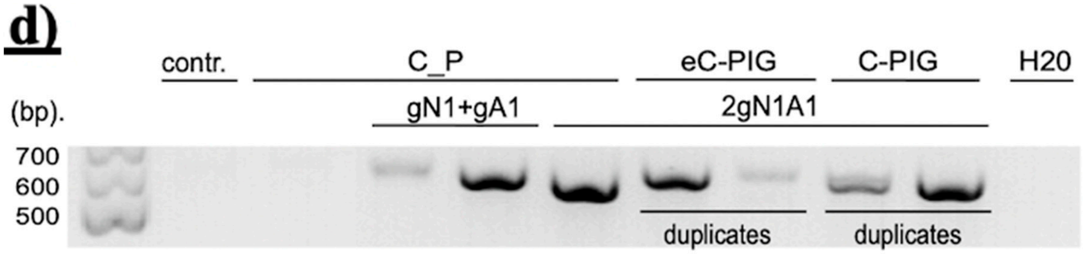

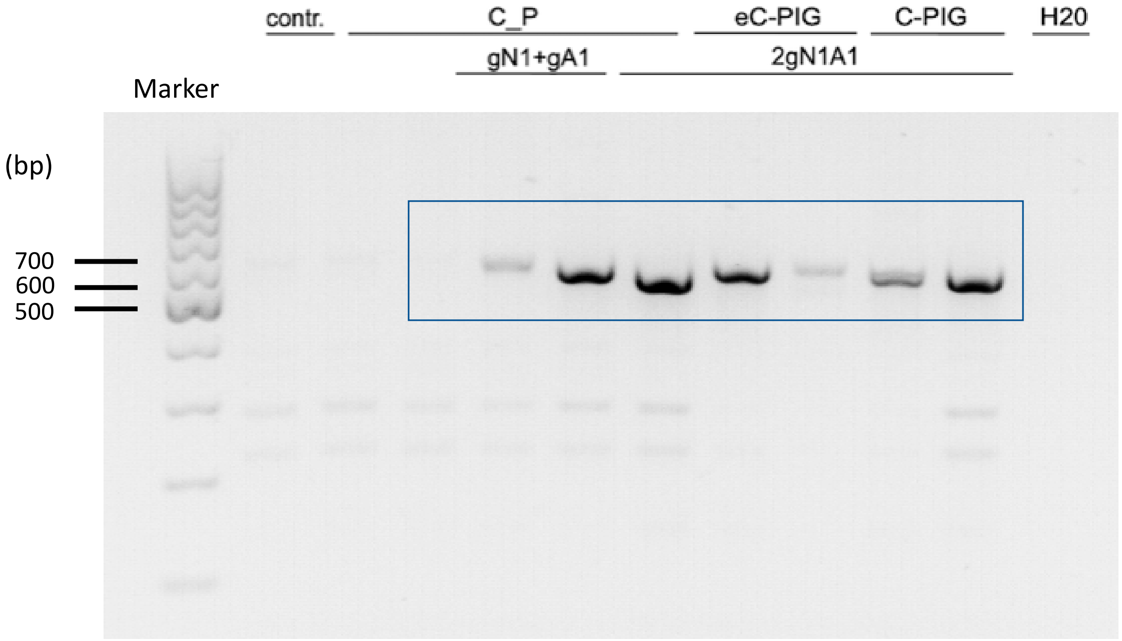

Supplement: Supplementary file 1 [file cancers-17-02226-s001.zip › cancers-3624185-supplementary.pdf]
